# Response to the comment on “Diagnostic value of a computer-assisted diagnosis system for the ultrasound features in thyroid nodules”

**DOI:** 10.20945/2359-4292-2024-0526

**Published:** 2025-02-24

**Authors:** Yiwei Wang

**Affiliations:** 1 Beijing Luhe Hospital, Capital Medical University, Ultrasound Department, Beijing, China

**DEAR EDITOR**,

We thank Dr. Ahmet Bozer for carefully reading our article titled “Diagnostic value of a computer-assisted diagnosis system for the ultrasound features in thyroid nodules” published in the *Archives of Endocrinology and Metabolism* (AE&M) and affirming the results of our research and providing constructive comments.

We present the findings of our retrospective study on still images, which were exclusively utilized for an initial investigation into the software’s capability. Between 2023 and 2024, our team prospectively collected dynamic images from patients with thyroid nodules, aiming to advance the software’s dynamic recognition capabilities. We are dedicated to swiftly and precisely transitioning from static to dynamic image recognition. Dynamic ultrasound images were included on the basis of the following criteria: visible thyroid nodules; visible maximal cross-sectional area of the nodule; and a 180-degree view of the nodule, with scans covering the central axis until the nodule was no longer visible. Each dynamic sequence lasted 30 seconds. Furthermore, all patients included in subsequent dynamic imaging analyses were prospectively enrolled (^1^,^2^).

Factors such as cervical lymph node assessment and vascularity are critical for stratifying the risk of malignancy. These methods significantly increase the diagnostic accuracy of computer-aided diagnosis (CAD) systems. Currently, we are investigating the value of the elasticity coefficient ratio of the nodule to the surrounding tissue and the multiparametric prediction of nodular traits. However, because some thyroid nodules have limited blood flow, high-quality color Doppler images are difficult to obtain. This limitation can affect the quality of machine learning models and, consequently, diagnostic accuracy. To address this, we are exploring various techniques, including energy Doppler and contrast-enhanced ultrasound with microbubble perfusion, to improve blood flow imaging (^3^). We also appreciate your suggestion to incorporate cervical lymph node characteristics. In future studies, we aim to conduct multimodal analyses that integrate peripheral lymph nodes, surrounding tissues (^4^), and other clinical features to better characterize thyroid nodules, thereby enhancing the performance of the CAD system in diagnosing thyroid nodule characteristics (^5^).

Thank you very much for your valuable comments. Your constructive feedback has played an essential role in shaping our subsequent research experiments.
